# Predictive value of early neutrophil-to-lymphocyte ratio and C-reactive protein in pediatric steroid-sensitive nephrotic syndrome

**DOI:** 10.3389/fneph.2025.1524231

**Published:** 2025-02-14

**Authors:** Gulinuer Maimaititusvn, Nilupaer Jvnaiti, Maierhaba Kulaixi, Fang Liu

**Affiliations:** ^1^ Department of Pediatrics, Kashi Prefecture Second People's Hospital, Kashgar, Xinjiang Uygur Autonomous Region, China; ^2^ Department of Pediatrics, Shanghai East Hospital, Tongji University, Shanghai, China; ^3^ School of Medicine, Tongji University, Shanghai, China

**Keywords:** pediatric steroid-sensitive nephrotic syndrome, NLR, CRP, relapse, adverse prognosis

## Abstract

**Objective:**

This study aims to investigate the predictive value of early neutrophil-to-lymphocyte ratio (NLR) and C-reactive protein (CRP) levels for relapse and adverse prognosis within one year in children diagnosed with steroid-sensitive nephrotic syndrome (SSNS).

**Methods:**

This study included a total of 145 pediatric patients diagnosed with steroid-sensitive nephrotic syndrome (SSNS) between January 2016 and December 2021. We collected early neutrophil-to-lymphocyte ratio (NLR) and C-reactive protein (CRP) levels, along with relevant clinical data, and conducted a one-year follow-up of the patients. Based on the follow-up outcomes, the patients were categorized into two groups: those who experienced a relapse and those who did not. We assessed the diagnostic and predictive value of NLR and CRP for relapse within one year using receiver operating characteristic (ROC) curve analysis and the Cox proportional hazards regression model.

**Results:**

After an average follow-up period of one year, 95 patients (65.52%) experienced relapse, while 50 patients (34.48%) did not. Significant differences were noted between the relapse and non-relapse groups regarding neutrophil-to-lymphocyte ratio (NLR), platelet-to-lymphocyte ratio (PLR), C-reactive protein (CRP), 24-hour urinary protein levels, age at onset, and parental education level (P < 0.05). Cox proportional hazards regression analysis identified age at onset, NLR, CRP, and 24-hour urinary protein levels as significant risk factors for relapse in patients with steroid-sensitive nephrotic syndrome (SSNS). Receiver operating characteristic (ROC) curve analysis for the combined prediction of relapse using NLR, CRP, and 24-hour urinary protein levels demonstrated good predictive value, with an area under the curve (AUC) of 0.858 (95% CI: 0.80–0.916, P < 0.001). Kaplan-Meier survival analysis indicated that patients with elevated NLR (≥ 2.90) and CRP (≥ 25.30) exhibited the highest relapse rates and shorter survival times. Further Cox proportional hazards analysis revealed that children in the high NLR and high CRP groups were at an increased risk of relapse, rehospitalization, infection, prolonged cumulative steroid use, renal insufficiency, secondary hypertension, and other adverse outcomes within one year.

**Conclusion:**

Early levels of Neutrophil-to-Lymphocyte Ratio (NLR) and C-Reactive Protein (CRP) demonstrate significant predictive value for relapse and adverse prognosis within one year in children with Steroid-Sensitive Nephrotic Syndrome (SSNS). These markers can serve as effective tools for auxiliary clinical assessment.

## 1 Introduction

Steroid-Sensitive Nephrotic Syndrome (SSNS) is a prevalent renal disorder in childhood, clinically characterized by significant proteinuria, hypoalbuminemia, hyperlipidemia, and edema. While the majority of affected children respond favorably to steroid therapy, a subset may experience relapses post-treatment, with some progressing to unfavorable outcomes, such as renal insufficiency. Therefore, identifying effective predictive markers is essential for early intervention and enhancing prognosis. Recent studies have indicated that the neutrophil-to-lymphocyte ratio (NLR) and C-reactive protein (CRP), both established inflammatory markers, are closely associated with the occurrence and progression of various diseases.

This study aims to investigate the potential utility of early NLR and CRP levels in predicting relapse and adverse outcomes within one year for children diagnosed with SSNS.

## 2 Materials and methods

### 2.1 Patient

This study focused on pediatric patients newly diagnosed with Primary Nephrotic Syndrome (PNS) who received treatment from pediatric physicians in the Kashgar region of Xinjiang between January 2016 and December 2021. The diagnosis adhered to the criteria established in the 2016 Guidelines for the Diagnosis and Treatment of Steroid-Sensitive, Relapsing/Dependent Nephrotic Syndrome in Children (1). Clinical features included significant proteinuria, defined as protein excretion of 40 mg/h/m², ≥1000 mg/m²/day, or a urinary protein-to-creatinine ratio of ≥2 mg/mg or 3+; hypoalbuminemia, indicated by a 24-hour urine protein exceeding 3.5 g and serum albumin levels below 30 g/L; edema; and hyperlipidemia, characterized by serum cholesterol levels greater than 5.7 mmol/L or 200 mg/dL.

The inclusion criteria for this study were as follows: (1) participants must have been hospitalized at our institution between January 2016 and December 2021, met the diagnostic criteria for PNS (1), received their first diagnosis of PNS without having initiated hormone therapy, and be aged 18 years or younger. (2) Exclusion of other forms of nephrotic syndrome, including congenital nephrotic syndrome, secondary nephrotic syndrome, Henoch-Schönlein purpura, hepatitis B, lupus, and other diseases associated with PNS. The exclusion criteria included: (1) Congenital nephrotic syndrome; (2) Secondary nephrotic syndrome; (3) Steroid-resistant PNS, encompassing both primary and delayed steroid resistance; (4) Patients with primary nephrotic syndrome who were confirmed to have IgA nephropathy via renal biopsy; (5) Loss to follow-up, a follow-up duration of less than one year, or incomplete clinical data;(6). The initial relapse is characterized by the presence of a clear active infection, which may manifest as symptoms such as fever, cough, and frequent urination accompanied by urgency due to bacterial infection. Laboratory tests reveal inflammatory indicators, including an elevated white blood cell count, increased levels of C-reactive protein, and positive results from blood or urine cultures.

### 2.2 Research methods

#### 2.2.1 Data collection

##### 2.2.1.1 Demographic and Clinical Information

The following data were collected: the child’s gender, ethnicity, age at disease onset, clinical classification (simple or nephritic type), residence (urban or rural), and parental educational background (employment status as farmers or unemployed).

##### 2.2.1.2 Initial Laboratory Assessments (Blood tests were taken before routine hormone treatment on the day of admission)

White blood cells (WBC), the neutrophil-to-lymphocyte ratio (NLR), the platelet-to-lymphocyte ratio (PLR), the systemic immune inflammation index (SII), liver function, kidney function, serum immunoglobulin levels, C-reactive protein (CRP), and the erythrocyte sedimentation rate are all important parameters. Additionally, a variety of bone metabolism markers, including alkaline phosphatase, serum osteocalcin (OC), and type I collagen carboxyl telopeptide β special sequence (β-CTX), are also relevant. 

##### 2.2.1.3 Relapse Evaluation

Data regarding relapse triggers, the number of relapse episodes, and the duration from remission (defined by the resolution of proteinuria) to relapse were recorded.

##### 2.2.1.4 Clinical Outcome Assessment

The duration required for proteinuria to resolve following the initiation of corticosteroid therapy was documented.

#### 2.2.2 Inpatient treatment protocol

##### 2.2.2.1 Initial Corticosteroid Therapy for PNS

Prednisone acetate was administered at a dosage of 2 mg/kg/day (maximum 60 mg/day) for a duration of 4 weeks. This initial phase was followed by an alternate-day dosing regimen for an additional 4 weeks. Subsequently, the dosage was tapered by 2.5-5.5 mg every 4 weeks until the medication was completely discontinued.

##### 2.2.2.2 Relapse Management

In the event of a relapse, treatment was reinstated at a dose of 2 mg/kg/day (up to 60 mg/day) for at least 3 consecutive days. Once proteinuria resolved or decreased to a trace level, the dose was tapered to 1.5 mg/kg (or 40 mg/m²) every other day for 4 weeks, during which the patient was closely monitored.

#### 2.2.3 Follow-up plan

##### 2.2.3.1 Follow-up Schedule

Patients were scheduled for follow-up visits at the outpatient clinic 2 and 4 weeks post-discharge, followed by evaluations every 3 months. After the discontinuation of corticosteroids, follow-ups were arranged every 6 months.

##### 2.2.3.2 Definition of Clinical Remission

Patients were considered to have achieved clinical remission if no relapse occurred within 1 year of initiating corticosteroid therapy. Subsequently, follow-up could be conducted via telephone.

##### 2.2.3.3 Follow-up Assessments

At each follow-up visit, vital signs, overall condition, medication adherence, adverse effects, and urinalysis results were documented. The total duration of follow-up was 24 months.

#### 2.2.4 Relapse criteria

##### 2.2.4.1 Definition of Relapse

Relapse was defined as the re-emergence of nephrotic-range proteinuria following partial or complete remission achieved through a 6-week course of standard corticosteroid therapy.

##### 2.2.4.2 Diagnostic Criteria

Relapse was confirmed if urine dipstick testing indicated ≥3+ protein for 3 consecutive days or if the urine protein-to-creatinine ratio (UPCR) exceeded 200 mg/mmol in a morning urine sample, with or without the presence of edema. ③Exclude patients with recurrence and active infection.

#### 2.2.5 Definition and evaluation of related adverse prognosis during follow-up

##### 2.2.5.1 Abnormal Bone Metabolism

The determination of abnormal bone metabolism primarily relies on the assessment of bone metabolism markers that exceed the normal reference range. This evaluation is conducted by integrating the patient’s clinical symptoms and imaging findings. The normal reference range is established based on test data from a substantial cohort of healthy individuals and relevant clinical guidelines. Clinically, manifestations such as bone pain (notably in the lower back and limb bones) and an increased susceptibility to fractures are indicative of potential abnormalities. Imaging assessments utilize dual-energy X-ray absorptiometry (DXA) to measure bone mineral density (BMD). A T-score lower than -1.0, when compared to the bone density of healthy young individuals of the same gender and ethnicity, suggests a reduction in bone mass. A T-score below -2.5 is classified as osteoporosis. These conditions serve as critical evidence for diagnosing abnormal bone metabolism.

##### 2.2.5.2 Renal Insufficiency

Renal insufficiency is primarily defined by the glomerular filtration rate (eGFR) and serum creatinine (Scr) levels. We utilized the Schwartz formula: k * height (cm)/serum creatinine (mg/dl) to estimate eGFR. A diagnosis of renal insufficiency is established when the eGFR is below 90 mL/min/1.73 m², a condition that must persist for a minimum of three months, while excluding other factors that may lead to a temporary decline in renal function, such as the recovery period from acute kidney injury or dehydration. 

##### 2.2.5.3 Secondary Hypertension

During the follow-up period for children with nephrotic syndrome, blood pressure was measured three times on different days without the administration of antihypertensive medications. Secondary hypertension is defined when the systolic blood pressure (SBP) and/or diastolic blood pressure (DBP) exceeds the 95th percentile for children of the same age, gender, and height. The extensive use of glucocorticoids is a common contributor to hypertension secondary to nephrotic syndrome in this population. To differentiate secondary hypertension from essential hypertension, we inquired about any family history of hypertension. Additionally, patients were questioned regarding hypertension-related symptoms, such as headaches, dizziness, and blurred vision, which may indicate that elevated blood pressure has resulted in target organ damage.

##### 2.2.5.4 Infection

When a patient develops a fever (body temperature ≥37.5°C) during the follow-up period, accompanied by local symptoms such as cough, sputum production, frequent urination, urgency, dysuria, and wound suppuration, the possibility of infection is initially considered. Based on this assessment, laboratory test results are further analyzed. Abnormalities in the white blood cell count and classification, such as an increased percentage of neutrophils or a decreased percentage of lymphocytes, suggest a bacterial infection. Conversely, an abnormal increase in lymphocyte count may indicate a possible viral infection. Additionally, elevated levels of C-reactive protein (CRP), typically defined as CRP > 10 mg/L, and elevated procalcitonin (PCT), with PCT > 0.05 ng/mL indicating a likely bacterial infection, further support the diagnosis. If microbiological examinations, including blood, sputum, and urine cultures, identify pathogenic bacteria, the patient is confirmed to be infected. During the follow-up process, patients are thoroughly questioned regarding the presence of the aforementioned infection-related symptoms, and details such as the onset and duration of symptoms are meticulously recorded. Simultaneously, patients are scheduled for necessary laboratory tests at the corresponding follow-up intervals, which include routine blood tests, CRP, PCT assessments, and microbiological cultures. The results of these tests are systematically documented in a specially designed case record form for subsequent analysis.

##### 2.2.5.5 Hospitalization standards and assessment

Patients experiencing exacerbations of illness due to infection-related diseases, such as respiratory failure resulting from severe lung infections requiring respiratory support or sepsis from urinary tract infections, may necessitate hospitalization. Additionally, relapses of steroid-sensitive nephrotic syndrome (SSNS), if applicable to the study, characterized by severe symptoms such as massive proteinuria (urinary protein/creatinine ratio > 300), significant edema affecting cardiopulmonary function, and hypoalbuminemia leading to thrombosis and other complications, require close monitoring and potential adjustments to the treatment plan in a hospital setting. Furthermore, patients who develop other serious complications related to the study disease, including severe deterioration of renal function or severe hypertensive crises that necessitate hospitalization, are also considered to meet the criteria for hospitalization.

We connect to the hospital’s hospitalization information management system to obtain relevant patient data, including length of stay, reason for hospitalization, primary diagnosis, and treatment processes during the hospital stay. Additionally, during patient follow-up, the hospitalization status is verified again to ensure data accuracy. After integrating this information, it is recorded in a specialized case record form to facilitate subsequent statistical analysis.

### 2.3 Statistical analysis

Statistical analyses were performed using SPSS version 27.0. For normally distributed continuous variables, data are presented as mean ± standard deviation (SD), with comparisons between two groups conducted using the t-test. For non-normally distributed data, the median and interquartile range (IQR) are reported, and group comparisons are made using non-parametric tests. Categorical data are expressed as counts and percentages, with differences between groups analyzed using the Chi-square (χ²) test. Univariate analysis and Cox proportional hazards analysis were utilized to identify risk factors associated with relapse. Receiver operating characteristic (ROC) curves were employed to determine optimal cut-off values, and survival curves were constructed accordingly. The Kaplan-Meier method was used to compare survival rates between groups, while the Cox proportional hazards regression model was applied to identify independent risk factors for relapse in children with primary neurological syndromes (PNS).

## 3 Result

### 3.1 Baseline characteristics of the two groups

Statistically significant differences were observed between the two groups regarding age at disease onset, parental education level, and the presence of hypertension (P<0.05). Furthermore, notable differences were identified between the groups in terms of neutrophil-to-lymphocyte ratio (NLR), platelet-to-lymphocyte ratio (PLR), 24-hour urinary protein excretion, and C-reactive protein (CRP) levels (P<0.05), as illustrated in **Table 1**.

**Table 1:** Comparison of baseline characteristics between the two groups [μ ± s, n(%), M(QR)].

Data	Relapse Group (n=95)	Non-Relapse Group (n=50)	Z/t//χ2	P-value
Age at onset (years)	6.10 ± 0.41	7.59 ± 0.34	0.143	0.009
Gender (Male)	55 (57.89%)	36 (72.00%)	2.789	0.095
Gender (Female)	40 (42.10%)	14 (28.00%)		
Parental education level (Farmers)	52 (54.73%)	18 (36.00%)	4.606	0.032
Non-farmers	43 (45.26%)	32 (64.00%)		
Hypertension	21 (22.10%)	0	5.345	<0.001
Nephritic syndrome	60 (63.15%)	25 (50.00%)	2.338	0.126
NLR	2.89 ± 0.22	1.09 ± 0.80	5.82	<0.001
PLR	173.03 ± 18.02	117.02 ± 17.44	2.00	0.045
PCT (ng/ml)	2.48 ± 2.10	0.35 ± 0.02	0.731	0.466
IL-6 (pg/mL)	7.41 ± 0.41	8.49 ± 1.34	0.143	0.381
PLT (*10^9/L)	435.80 ± 15.38	396.40 ± 21.94	-0.878	0.193
IgG (g/l)	5.17 ± 0.45	4.72 ± 0.54	-0.56	0.575
IgM (g/l)	1.62 ± 0.75	1.80 ± 0.13	-1.101	0.271
24hUPT (g/24h)	3.33 ± 0.26	2.49 ± 0.31	-2.086	0.037
CRP (ng/ml)	29.61 ± 2.88	17.96 ± 4.20	2.551	0.012
TG (mmol/L)	2.54 ± 0.14	2.53 ± 0.27	-0.066	0.948
TC (mmol/L)	9.65 ± 0.38	8.60 ± 0.52	-1.706	0.088
HDL-C (mmol/L)	1.51 ± 0.06	1.68 ± 0.23	-0.064	0.337
LDL-C (mmol/L)	6.27 ± 0.29	5.47 ± 0.37	-1.319	0.187
ESR (mm/h)	52.71 ± 1.71	48.36 ± 3.14	-1.511	0.131
Cr (umol/L)	39.84 ± 4.15	54.14 ± 12.52	-1.229	0.221
BUN (mmol/L)	5.63 ± 0.46	7.41 ± 0.96	-1.719	0.088
UA (umol/L)	294.44 ± 10.21	334.88 ± 17.81	-1.94	0.054
AlB (g/L)	19.34 ± 1.00	19.67 ± 1.33	-0.12	0.903

NLR, Neutrophil-to-Lymphocyte Ratio; PLR, Platelet-to-Lymphocyte Ratio; PCT, Procalcitonin (ng/ml); IL-6, Interleukin-6 (pg/mL); PLT, Platelet Count (or Platelets) (*10^9/L); 24hUPT, 24hour urinary protein (g/24h); CRP, C-reactive protein (ng/ml); TG, Triglycerides (mmol/L); TC, otal Cholesterol (mmol/L); HDL-C, High-Density Lipoprotein Cholesterol (mmol/L); LDL-C, Low-Density Lipoprotein Cholesterol (mmol/L); ESR, Erythrocyte Sedimentation Rate (mm/h); Cr, Creatinine (umol/L); BUN, Blood Urea Nitrogen (BUN); UA, Uric acid (umol/L); ALB, Albumin (g/L).

### 3.2 Analysis of risk factors for relapse

Patients were classified into different age groups: the lower age group (≤5 years old), the middle age group (6-10 years old), and the older age group (>10 years old). Subsequently, we constructed a Cox proportional hazards regression model that included age, gender, NLR, and other relevant factors. The results of the multivariate Cox regression analysis indicated a Chi-square value of 186.352 (P < 0.001), demonstrating that NLR, time to first urine protein conversion, CRP, and the 24-hour urine egg quantification value are significant risk factors for the first recurrence, as shown in **Table 2**.

**Table 2:** Cox regression analysis of risk factors for relapse.

	B	SE	Wald	P-value	Exp (B)	95.0% CI
Age of onset (younger age group ≤ 5years)	-0.481	0.328	2.160	0.142	0.618	(0.325, 1.174)
Middle age group (6-10 years old)	-0.549	0.290	3.594	0.058	0.578	(0.327, 1.019)
Older age group (≥10 years)	–	–	3.677	0.159	–	–
gender	-0.190	-0.243	0.610	0.435	0.827	(0.514, 1.332)
Parental education level (Farmers)	-0.186	0.245	0.575	0.448	0.831	(0.514, 1.342)
Hypertension	0.666	0.285	5.449	0.202	1.946	(1.113, 3.404)
Time to first negative urine protein test (weeks)	1.130	0.109	108.205	0.001	3.094	(2.501, 3.828)
NLR	0.286	0.048	35.328	0.001	1.332	(1.212, 1.463)
PLR	0.000	0.005	0.002	0.969	1.000	(0.991, 1.009)
CRP(ng/ml)	0.009	0.004	5.561	0.010	1.009	(1.002, 1.017)
24hUPT(g/24h)	0.103	0.043	5.748	0.018	1.108	(1.019, 1.205)

### 3.3 ROC curve analysis

**Figure 1:**
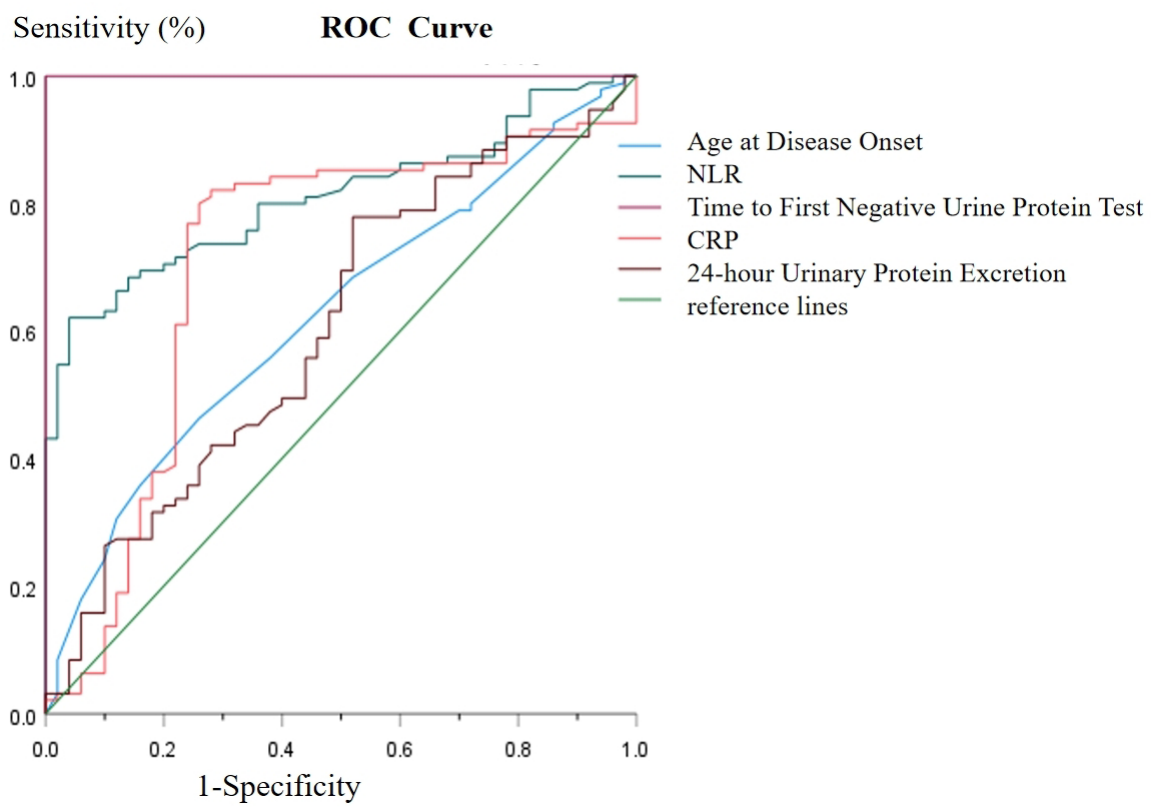
ROC curve of clinical biomarkers for combined to predict PNS relapse.

**Figure 2:**
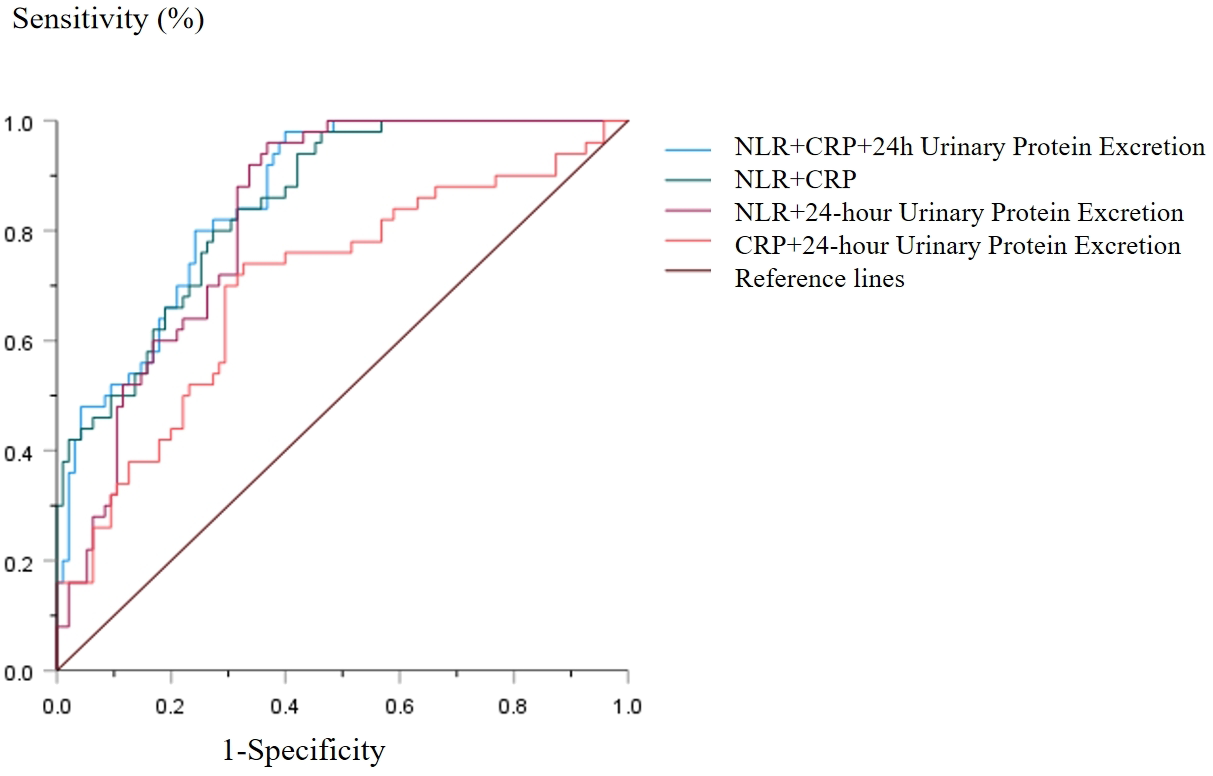
ROC curve of clinical biomarkers for combined to predict PNS relapse.

Receiver Operating Characteristic (ROC) curves were generated to evaluate the predictive value of several factors, including age at disease onset, neutrophil-to-lymphocyte ratio (NLR), C-reactive protein (CRP), time to the first negative urine protein test, and 24-hour urinary protein excretion, in relation to relapse probability. The findings indicated that NLR exhibited an Area Under the Curve (AUC) of 0.810 (95% CI: 0.742–0.878, P < 0.001), with a sensitivity of 62.10%, specificity of 92.90%, and a Youden index of 0.58. In comparison, CRP demonstrated an AUC of 0.708, with a Sensitivity 80.0%, specificity 73.50%, Youden index 0.535, corresponding CRP cutoff value: 20.30ng/ml. Additionally, 24-hour urinary protein excretion had an AUC of 0.606 (95% CI: 0.508–0.703, P = 0.037), with a sensitivity of 77.90%, specificity of 46.90%, and a Youden index of 0.203. Age at disease onset yielded an AUC of 0.626 (95% CI: 0.533–0.719, P = 0.013), with a sensitivity of 46.30%, specificity of 74.00%, and a Youden index of 0.248 (as illustrated in **Figures 1**, **2**, **Tables 3**, **4**). The combined predictive value of NLR, CRP, and 24-hour urinary protein excretion resulted in an AUC of 0.858 (95% CI: 0.800–0.916, P < 0.001), with a sensitivity of 98.00%, specificity of 60.00%, and a Youden index of 0.58. Furthermore, the combination of NLR and CRP alone produced an AUC of 0.849 (95% CI: 0.788–0.910, P < 0.001), with a sensitivity of 80.00%, specificity of 72.60%, and a Youden index of 0.526. Collectively, these combined variables demonstrated a strong predictive value for predicting PNS relapse within one year.

**Table 3:** Comparison of predictive values of clinical indicators for PNS relapse.

	AUC	Sensitivity (%)	Specificity (%)	Max Youden Index	Kappa Value	CI (95%)	P-value
NLR	0.810	62.10	92.90	0.58	1.97	(0.742, 0.878)	<0.001
24hUPT (g/24h)	0.606	77.90	46.90	0.203	7.50	(0.508, 0.703)	0.037
CRP	0.708	80.00	73.50	0.535	10.27	(0.611, 0.806)	<0.001
Age at Onset (years)	0.626	46.30	74.00	0.248	7.50	(0.533, 0.719)	0.013
Time to First Negative Urine Protein Test (weeks)	1.00	100.00	100.00	1	2	(0.298, 0.481)	<0.001

**Table 4:** Comparison of combined predictive values of clinical indicators.

	AUC	Sensitivity (%)	Specificity (%)	Max Youden Index	CI (95%)	P-value
NLR + CRP + 24hUPT	0.858	98.00	60.00	0.58	(0.800, 0.916)	<0.001
NLR+CRP	0.849	80.00	72.60	0.526	(0.788, 0.910)	<0.001
NLR + 24hUPT	0.827	96.00	63.20	0.592	(0.762, 0.891)	<0.001
CRP + 24hUPT	0.701	74.00	67.40	0.414	(0.608, 0.793)	<0.001

### 3.4 Comparison of general characteristics and routine biochemical indicators between high NLR and low NLR groups

Based on the median Neutrophil-to-Lymphocyte Ratio (NLR), patients were categorized into a high NLR group (≥ 2.90, n = 39) and a low NLR group (0.05). However, the high NLR group demonstrated significantly higher rates of 1-year relapse, re-hospitalization, infection frequency, secondary hypertension, renal insufficiency, and cumulative steroid use duration compared to the low NLR group, with all differences being statistically significant (P < 0.05). For further details, please refer to **Table 5**.

**Table 5:** Comparison of baseline characteristics between patients with high NLR (≥ 2.90) and low NLR (< 2.90) groups [μ ± SD, n (%), M (QR)].

Clinical Data	NLR ≥ 2.90 (n=39)	NLR < 2.90 (n=106)	z/t	P-Value
Age (years)	8.54 ± 0.50	7.56 ± 0.30	1.468	0.078
Gender (male)	22 (56.41%)	66 (62.26%)	0.41	0.522
Recurrence frequency	3.23 ± 0.13	1.43 ± 0.14	7.242	<0.001
Cumulative steroid use (weeks)	28.21 ± 0.77	17.93 ± 0.79	7.541	<0.001
Hospitalization frequency	3.21 ± 0.13	1.43 ± 0.14	7.118	<0.001
Infection frequency	2.87 ± 0.12	1.35 ± 0.13	6.702	<0.001
Bone metabolism abnormalities	11 (28.20%)	10 (9.43%)	2.895	0.089
Renal insufficiency	10 (25.64%)	7 (6.60%)	9.984	0.002
Secondary hypertension	16 (41.02%)	12 (11.32%)	16.145	<0.001
Recurrence	38 (97.44%)	55 (51.89)	25.717	<0.001

### 3.5 Comparison of general characteristics and routine biochemical indicators between high CRP and low CRP groups

Patients were categorized into two groups based on the median C-reactive protein (CRP) level: the high CRP group (≥25.30 mg/L, n=63) and the low CRP group (<25.30 mg/L, n=82). No statistically significant differences were observed between the two groups regarding age or gender. However, the high CRP group demonstrated significantly elevated rates of recurrence within one year, rehospitalization, infection frequency, secondary hypertension, renal insufficiency, and cumulative steroid use compared to the low CRP group. All these differences were statistically significant (P < 0.05), as detailed in **Table 6**.

**Table 6:** Comparison of baseline characteristics between high CRP (≥25.30) and low CRP (<25.30) groups [μ ± SD, n (%), M (QR)].

Clinical Data	CRP ≥ 25.30 (n=63)	CRP < 25.30 (n=82)	z/t	P-Value
Age (years)	6.91 ± 0.41	7.22 ± 0.35	-0.583	0.561
Gender (male)	34 (53.90%)	54 (65.85%)	0.546	0.068
Recurrence frequency	2.41 ± 0.18	1.54 ± 0.17	3.520	<0.001
Cumulative steroid use (weeks)	35.12 ± 1.78	27.08 ± 1.71	-3.169	0.002
Hospitalization frequency	2.41 ± 0.18	1.52 ± 0.17	3.580	<0.001
Infection frequency	2.24 ± 0.16	1.39 ± 0.15	3.820	<0.001
Bone metabolism abnormalities	13 (20.63%)	8 (9.75%)	3.404	0.065
Renal insufficiency	10 (15.87%)	10 (12.19%)	0.405	0.524
Secondary hypertension	19 (30.16%)	9 (10.98%)	8.414	0.004
Recurrence	54 (85.71)	41 (50.00%)	20.115	<0.001

### 3.6 Survival curves for NLR and CRP in predicting postoperative recurrence

**Figure 3:**
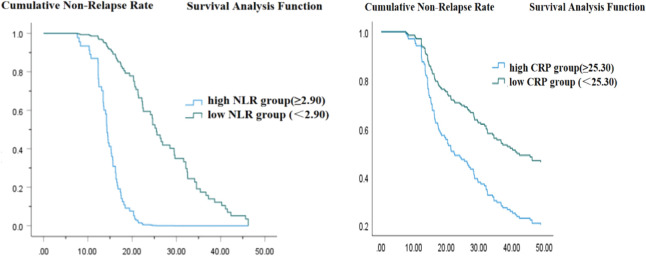
**(A)** Time from urine protein negative conversion to first relapse (weeks). **(B)** Time from urine protein negative conversion to first relapse (weeks).

The Kaplan-Meier analysis demonstrated that patients with a high neutrophil-to-lymphocyte ratio (NLR) experienced a significantly shorter survival time compared to those with a low NLR (Log Rank < 0.001, **Figure 3A**). Likewise, patients classified in the high C-reactive protein (CRP) group exhibited a reduced survival time relative to those in the low CRP group (Log Rank < 0.001, **Figure 3B**).

### 3.7 Determination of prognostic risk factors

Multivariate Cox regression analysis revealed that children with Primary Nephrotic Syndrome (PNS) exhibiting a Neutrophil-to-Lymphocyte Ratio (NLR) of ≥ 2.90 and C-reactive Protein (CRP) levels of ≥ 20.30 ng/ml faced an elevated risk of recurrence within one year, as well as complications such as bone metabolism abnormalities, renal insufficiency, and secondary hypertension. In comparison to children with low NLR and low CRP levels, those classified in the high NLR and CRP groups experienced a longer duration of cumulative steroid use, more frequent hospitalizations, and a greater incidence of infections (P < 0.05), as illustrated in **Table 7**.

**Table 7:** Cox regression analysis of prognostic risk factors.

Clinical Data	HR (95% CI )	P-Value	HR (95% CI )	P-Value
Cumulative steroid use (≥ 20.70 weeks)	2.120 (1.395, 3.222)	<0.001	1.594 (1.050, 2.421)	0.029
Hospitalization frequency (≥ 2 times)	1.893 (1.258, 2.849)	<0.001	0.663 (0.443, 0.992)	0.046
Infection frequency (≥ 3 times)	3.624 (2.058, 6.381)	<0.001	0.487 (0.274, 0.864)	0.012
Bone metabolism abnormalities	2.990 (1.27, 7.040)	0.012	0.473 (0.196, 1.41)	0.095
Renal insufficiency	4.077 (1.667, 9.974)	0.002	0.768 (0.320, 1.846)	0.556
Secondary hypertension	3.624 (1.714, 7.660)	<0.001	0.364 (0.165, 0.804)	0.012
Recurrence	1.893 (1.258, 2.849)	<0.001	0.635 (0.424, 0.952)	0.028
Recurrence frequency (≥ 3 times)	3.953 (2.297, 6.803)	<0.001	1.754 (1.023, 3.009)	0.041

## 4 Discussion

Primary Nephrotic Syndrome (PNS) is a condition characterized by glomerular filtration dysfunction arising from various etiologies, which leads to a spectrum of pathophysiological alterations and metabolic disturbances (1). The global incidence of PNS in children varies between 1.15 and 16.90 per 100,000 individuals. Notably, a multicenter study conducted in China indicated that PNS accounts for 20% of urinary system diseases among hospitalized children during the same timeframe (2, 3). Glucocorticoid therapy serves as the first-line treatment for newly diagnosed childhood nephrotic syndrome, with approximately 80% to 90% of children responding favorably and achieving negative urine protein levels within 4 to 8 weeks of short-term treatment. Nonetheless, 50% to 70% of these children may experience recurrences, with 30% to 40% classified as frequent relapses. Such recurrences can lead to the gradual progression of kidney disease and, in some instances, chronic kidney disease or renal failure (4, 5, 7). A comprehensive meta-analysis by Veltkamp et al. (6) indicated that, between 1945 and 2011, the overall incidence of PNS in children remained relatively stable, while the recurrence rate decreased from 87.4% to 66.2%. Despite advancements in medical technology contributing to this decline in recurrence rates, the risk of recurrence persists, with notable regional variations. Clinically, over 85% of PNS cases in children are categorized as minimal change disease, with the majority of patients demonstrating steroid responsiveness. However, in China, 80% to 90% of children experience recurrences, and 25% to 43% progress to frequent relapses or steroid dependence. The occurrence of recurrences heightens the risk of complications such as sepsis, thrombosis, dyslipidemia, and malnutrition. Furthermore, prolonged high-dose steroid therapy and the administration of immunosuppressants are significantly associated with adverse effects, including avascular necrosis of the hip, hypertension, diabetes, and behavioral disorders.

The peak incidence of Nephrotic Syndrome (NS) is generally observed between the ages of 2 and 5, with a higher prevalence among male children. International studies indicate that the likelihood of disease recurrence correlates with the age at initial onset, suggesting that younger children are at greater risk for recurrence. Specifically, those with an onset age of 4 years or younger are classified as being at high risk for frequent relapses (10, 11). Conversely, other studies (8, 9, 12) have reported no significant correlation between the age of onset and the rate of recurrence. In our study, the median age of the 145 children was 7 years, with a range from 13 months to 14.75 years and a mean age of 6.04 years. Notably, children under 7 years old comprised 61.38% of the cases. The male-to-female ratio was 1.54:1. Consistent with previous research, our findings demonstrate that children in the recurrence group were younger at the time of onset, and the age of onset emerged as a significant risk factor for recurrence within one year (P < 0.05).

This study identified the time to initial urine protein remission as a significant risk factor for recurrence in children with primary nephrotic syndrome (PNS), as demonstrated by Cox proportional hazards survival analysis (P < 0.001). The mean time to urine protein remission for the recurrence group was 3.38 ± 0.09 weeks, in contrast to 1.30 ± 0.37 weeks for the non-recurrence group, indicating a statistically significant difference (P < 0.001). Nakanishi et al. (13) suggested that patients with a time to initial urine protein remission exceeding nine days were at an increased risk of experiencing frequent recurrences within six months. Similarly, Dakshayani et al. (14) established that the timing of the first recurrence serves as a risk factor for subsequent frequent recurrences. Kirtisudha (15) also noted that children who experienced their first relapse within six months were more likely to develop steroid dependence. In an eight-week steroid treatment study involving 32 children with frequently relapsing nephrotic syndrome (FRNS), Nakanishi (13) reported that all patients had their first relapse within six months, and Cox regression analysis identified recurrence within this timeframe post-remission as a risk factor for FRNS. Takeda et al. (12), in a one-year study of 121 patients, found that recurrence within the first year of onset significantly increased the risk of future recurrences, while the number of recurrences and duration of steroid therapy had a minimal effect on the subsequent disease course. As early as 1982, the International Study of Kidney Disease in Children (ISKDC) (16) proposed that the frequency of recurrences within six months correlates with future recurrences or frequent relapses, underscoring the importance of the timing of recurrence as a predictor of frequent relapse. The variability in the time to urine protein remission across studies may be attributable to differences in steroid treatment regimens and individual patient characteristics. These findings suggest that an early recurrence following remission is associated with a heightened likelihood of future relapses, whereas prolonged remission is indicative of a more favorable prognosis.

Proteinuria is widely recognized as a critical marker for predicting disease progression and adverse outcomes in kidney diseases (17). The quantification of urine protein over a 24-hour period at the onset of the disease can significantly influence the risk of nephrotic syndrome recurrence, mediated by various factors such as renal pathological changes, immune responses, treatment modalities, and lifestyle choices. Firstly, the pathological classifications of nephrotic syndrome are varied, and the initial urine protein quantification may serve as an indicator of the severity of renal damage. For instance, conditions like membranous nephropathy and focal segmental glomerulosclerosis are associated with an elevated risk of recurrence due to their inherent pathological characteristics. Secondly, nephrotic syndrome is intimately linked to abnormalities in the immune system. During the initial onset, the deposition of immune complexes on the glomerular basement membrane, activated by the complement system, results in significant proteinuria by enhancing glomerular permeability and further compromising the filtration barrier. If immune dysregulation is not adequately managed, triggers such as infections or physical stress can elicit additional immune responses, potentially leading to recurrence. Furthermore, elevated proteinuria at the initial onset may signify an activated state of immune cells, wherein immune imbalance could increase the likelihood of recurrence. However, some studies have indicated no significant correlation between 24-hour urine protein levels and the progression of glomerular diseases or the degree of tubular damage (18, 19, 27). This study utilized multivariate Cox regression analysis, revealing that early quantification of 24-hour urine protein is a significant risk factor for the first recurrence of nephrotic syndrome within one year in children (Model: Chi-square: 198.422, P 3.5g/24h) typically signifies a considerable breakdown of barrier function, frequently associated with significant tubular fibrosis or even necrosis. Sun Liangzhong et al. (20) observed that children with persistent proteinuria are at an elevated risk of renal impairment compared to those exhibiting intermittent proteinuria. Consequently, relying solely on a single 24-hour urine protein measurement to evaluate overall disease progression may be quite limited.

The neutrophil-to-lymphocyte ratio (NLR) is an inexpensive, cost-effective, and clinically accessible inflammatory marker (24). It is calculated by dividing the neutrophil count by the lymphocyte count. Neutrophils play a critical role in the inflammatory response, while lymphocytes are essential for immune regulation. An elevated NLR signifies a relative increase in neutrophils and a relative decrease in lymphocytes, indicating an intensified inflammatory response and potential immune dysfunction. Previous studies have demonstrated that NLR is associated with various inflammatory conditions, including cancer, cirrhosis, antineutrophil cytoplasmic antibody (ANCA)-associated vasculitis, and acute coronary syndromes (21). Further research has established a link between NLR and several adverse cardiovascular outcomes, such as decompensated heart failure, postoperative recurrence of atrial fibrillation, acute coronary syndrome, and sudden cardiac death (25, 26). Recent studies have also investigated the relationship between peripheral blood markers and kidney disease, identifying NLR as a prognostic indicator for renal disease outcomes. A high NLR (>3) has been correlated with renal function deterioration and the progression to advanced chronic kidney disease (CKD) (22). Toraman et al. (23) evaluated 54 patients and discovered that the platelet-to-lymphocyte ratio (PLR) could indicate the severity of crescentic glomerulonephritis during its acute phase, while NLR could be utilized to assess the extent of diabetic nephropathy and screen for kidney disease. In a study of 99 adult cases of idiopathic membranous nephropathy, Tsai et al. (21) found that NLR >3.34 (HR=3.30, P14.48 (HR=2.54, P=0.003) were associated with poor renal outcomes. Our study demonstrated that the neutrophil-to-lymphocyte ratio (NLR) levels at initial diagnosis were significantly higher in the recurrence group compared to the non-recurrence group, indicating a more severe inflammatory response among those with recurrence. This suggests that NLR may hold potential as a predictive biomarker for early relapse in steroid-sensitive nephrotic syndrome (SSNS). In patients with SSNS, inflammation may contribute to the onset and progression of the disease, resulting in damage to the glomerular basement membrane and the subsequent development of proteinuria. Elevated NLR levels could signify a heightened inflammatory response and immune dysregulation, which may increase the risk of recurrence. Additionally, C-reactive protein (CRP), an acute-phase protein that rises rapidly in response to infections, trauma, or other stimuli, is also relevant in this context. In children diagnosed with SSNS, immune dysregulation and inflammatory responses are evident, and the presence of elevated NLR and CRP levels may correlate with disease onset and progression.

This study employed multifactor Cox regression analysis to identify prognostic risk factors and found that a neutrophil-to-lymphocyte ratio (NLR) of ≥ 2.90 and a C-reactive protein (CRP) level of ≥ 20.30 ng/ml are significant risk factors for poor prognosis in children with primary nephrotic syndrome (PNS) within one year. These findings indicate that inflammation-related markers are closely associated with adverse outcomes, including recurrence, abnormal bone metabolism, renal insufficiency, and secondary hypertension in this population. During the one-year follow-up period, 20 cases (13.79%) developed chronic renal insufficiency, predominantly among children aged 6 to 10 years, with an average age of 6.15 years. In terms of gender distribution, there were 11 male and 9 female patients. Regarding clinical indicators, the average 24-hour urine protein quantification at the onset was 4.29 g, the serum albumin level was notably low, averaging 19.79 g/L, and the average serum creatinine level was 65.54 μmol/L. Among these patients, the proportion of those in the high NLR group was higher, comprising 15 cases (75%). Throughout the treatment process, a series of targeted measures were implemented. To manage the condition of SSNS, the immunosuppressive regimen has been modified, and protective measures for renal function have been enhanced. This includes the use of angiotensin-converting enzyme inhibitors (ACEIs) or angiotensin II receptor blockers (ARBs) to reduce proteinuria, safeguard renal function, and closely monitor changes in renal function indicators. From a pathophysiological perspective, the inflammatory response is pivotal in the onset and progression of nephrotic syndrome. Elevated levels of the neutrophil-to-lymphocyte ratio (NLR) suggest that the body may be experiencing a persistent inflammatory state. An increase in neutrophils coupled with a relative decrease in lymphocytes can disrupt immune regulation, thereby facilitating the development of renal lesions and the emergence of complications (28, 29). Similarly, C-reactive protein (CRP) is an acute phase response protein, and its elevation indicates the degree of inflammation present in the body. Ongoing inflammatory stimuli may inflict damage on kidney tissue and heighten the risk of other complications, such as chronic renal insufficiency and secondary hypertension.

This study found that, compared to children with primary nephrotic syndrome (PNS) exhibiting low neutrophil-to-lymphocyte ratio (NLR) and low C-reactive protein (CRP), those with high NLR and high CRP experienced longer cumulative steroid use, as well as increased rates of hospitalizations and infections (P < 0.05). This disparity underscores the detrimental impact of inflammatory status on disease progression. Hormones are a primary treatment modality for PNS. However, prolonged hormone use not only heightens the risk of adverse reactions in children, such as growth retardation and osteoporosis, but also reflects the refractory nature of the disease (30). Frequent hospitalizations and infections may create a vicious cycle, where infections exacerbate the inflammatory response and further compromise kidney function, ultimately leading to disease recurrence. Deteriorating kidney function increases children’s susceptibility to infections (31). These research findings hold significant clinical implications. Healthcare professionals can utilize NLR and CRP as straightforward and effective indicators for assessing the prognosis of children with PNS. In clinical practice, children with NLR ≥ 2.90 and CRP ≥ 20.30 ng/ml should be closely monitored for any changes in their condition, which includes regular assessments of renal function, bone metabolism indicators, and blood pressure. Additionally, proactive measures should be implemented to prevent infections and judiciously adjust hormone treatment plans to lower recurrence rates and minimize complications. For instance, the concurrent use of immunomodulators may be considered during treatment to enhance the body’s immune status and mitigate inflammatory responses (32).

This study has several limitations that must be acknowledged. Firstly, the small sample size may compromise the accuracy and reliability of the findings. Secondly, as a single-center retrospective study, there is a risk of selection bias. Moreover, since the Neutrophil-to-Lymphocyte Ratio (NLR) is derived from the ratio of neutrophils to lymphocytes, variations in either cell type can significantly affect the NLR value. This necessitates careful optimization of measurement timing and consideration of potential confounding factors. Additionally, the NLR levels observed in the recurrence group showed significant fluctuations throughout the treatment period, remaining consistently higher than those in the non-recurrence group. Further research is essential to investigate the relationship between dynamic changes in NLR and the relapse of Steroid-Sensitive Nephrotic Syndrome (SSNS). Future studies should aim to include multicenter, large-sample prospective designs to validate the predictive value of NLR and C-reactive protein (CRP) in SSNS recurrence among children and to elucidate the underlying mechanisms. Monitoring fluctuations in NLR could facilitate the early detection of disease instability, thereby providing a foundation for the timely adjustment of treatment strategies.

## 5 Conclusion

Early neutrophil-to-lymphocyte ratio (NLR) and C-reactive protein (CRP) values are significantly predictive of relapse and poor prognosis within one year for children with steroid-sensitive nephrotic syndrome (SSNS). Clinicians can evaluate disease progression and prognosis by monitoring these early NLR and CRP levels, which may serve as a reference for developing individualized treatment plans. Future research should focus on expanding the sample size and conducting multicenter studies that incorporate stratified analyses of NLR and other relevant indicators to validate their predictive value and identify more effective prognostic markers.

## Data Availability

The original contributions presented in the study are included in the article/supplementary material. Further inquiries can be directed to the corresponding author.
